# Skin dose during radiotherapy: a summary and general estimation technique

**DOI:** 10.1120/jacmp.v13i3.3734

**Published:** 2012-05-10

**Authors:** Stephen F. Kry, Susan A. Smith, Rita Weathers, Marilyn Stovall

**Affiliations:** ^1^ Department of Radiation Physics The University of Texas M. D. Anderson Cancer Center Houston TX USA

**Keywords:** skin dose, basal cell carcinoma, radiotherapy, surface dose, skin cancer

## Abstract

The skin dose associated with radiotherapy may be of interest for clinical evaluation or investigating the risk of late effects. However, skin dose is not intuitive and is difficult to measure. Our objectives were to develop and evaluate a general estimation technique for skin dose based on treatment parameters. The literature on skin dose was supplemented with measurements and Monte Carlo simulations. Using all available data, a general dosimetry system was developed (in the form of a series of equations) to estimate skin dose based on treatment parameters including field size, the presence of a block tray, and obliquity of the treatment field. For out‐of‐field locations, the distance from the field edge was also considered. This dosimetry system was then compared to TLD measurements made on the surface of a phantom. As compared to measurements, the general dosimetry system was able to predict skin dose within, on average, 21% of the local dose (4% of the $D_{\max}$ dose). Skin dose for patients receiving radiotherapy can be estimated with reasonable accuracy using a set of general rules and equations.

PACS numbers: 87.53.‐j, 87.53.Bn, 87.55.ne

## I. INTRODUCTION

The skin is at risk during radiotherapy for such effects as erythema, desquamation, and necrosis.^(^ ^1^ ^,^ ^2^ ^)^ Epidemiologic studies have also found an association between radiation therapy and basal‐cell carcinoma (BCC) induction.^(^ ^3^ ^–^ ^8^ ^)^ However, skin dosimetry is a challenge. Not only is there is a lack of a comprehensive overview of skin doses associated with radiotherapy in the literature, skin doses are also not generally intuitive compared to doses throughout the rest of the body, differing both in magnitude and in dependence on treatment parameters. Dosimetry for the skin is further complicated by the different structures of the skin (including basal and dermal layers), the depth of which varies not only between patients, but also between locations on a given patient. The International Commission on Radiological Protection recommends assessing the skin dose at a depth of 0.07 mm (basal layer),^(^ ^9^ ^,^ ^10^ ^)^ while the dermal layer may be assessed at 1.0 mm.^(^ ^11^ ^)^ The dose to the basal layer (0.07 mm) is often used interchangeably with surface dose.^(^ ^12^ ^–^ ^14^ ^)^

Although the skin dose may be calculated with the treatment planning system, generally within ±25% accuracy,^(^ ^15^ ^–^ ^17^ ^)^ this approach requires CT images and a calculated treatment plan. As such, it is not always a feasible option, for example when an initial skin dose estimate is sought, or when conducting retrospective epidemiology studies (e.g., evaluating BCC induction) for which CT scans are not available.

A skin dose estimation technique is therefore of general interest. Such a method was proposed by Lamb and Blake^(^ ^18^ ^)^ to estimate the skin dose on the central axis as a function of treatment parameters. However, their model assessed only 6 and 10 MV beams, and is limited to the central axis of the beam. Therefore, this study develops, presents, and evaluates a more general set of guidelines for estimating the skin dose for a wider range of energies and locations on the patient. To facilitate model development, this work also presents a literature summary on surface doses, as well as our own supplemental measurements and Monte Carlo simulations. This skin dose model was developed to determine skin doses for epidemiologic studies of BCCs, but can easily be applied to clinical situations in which skin dose is of interest.

Throughout this work, percent differences may be either absolute or relative (also called local). Absolute refer to percent differences relative to the $D_{\max}$ dose. Ten percent versus 20% of the $D_{\max}$ dose is a 10% absolute difference or a 100% relative/local difference.

## II. SKIN DOSE OVERVIEW

### A. Literature review

#### A.1 Energy and field size

**Figure 1:**
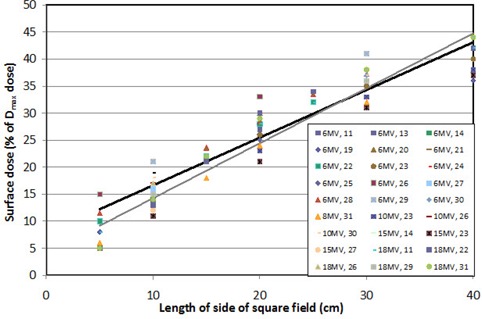
Surface dose (as a percentage of the $D_{\max}$ dose) from open megavoltage fields (6 MV−18 MV) as a function of the size of the edge of the equivalent square field. Included is a linear best‐fit line for high‐energy (15−18 MV) beams (thin line) as well as for low‐energy (6−10 MV) beams (thick line). Data series are described by the beam energy as well as the corresponding reference number.

**Figure 2:**
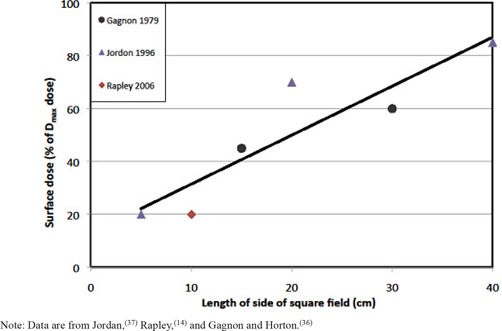
Surface dose (as a percentage of the $D_{\max}$ dose) from open cobalt fields as a function of the size of the edge of the equivalent square field. Included is a linear best‐fit line (Eq. (3)). Note: Data are from Jordan,^(^ ^37^ ^)^ Rapley,^(^ ^14^ ^)^ and Gagnon and Horton.^(^ ^36^ ^)^

Dose to the entrance surface (on the central axis of open megavoltage treatment fields with normal beam incidence and 100 cm source‐to‐surface distances [SSDs]) is between ~10% and ~45% of the $D_{\max}$ dose (also known historically as the given dose or entrance dose) (Fig. 1). These data include numerous makes and models of accelerator, although this figure is primarily based on Varian 2100/2300 accelerators; no obvious distinction was seen between different accelerators, although this observation was highly confounded by the differences in methodologies between different studies. Universally, the surface dose increases linearly with field size. While many studies have found a reduced surface dose at higher MV energies,^(^ ^23^ ^,^ ^26^ ^,^ ^29^ ^,^ ^32^ ^)^ others have not, particularly for large field sizes,^(^ ^11^ ^,^ ^31^ ^,^ ^33^ ^–^ ^35^ ^)^ as demonstrated with the low‐energy (6−10 MV) and high‐energy (15−18 MV) best‐fit lines in Fig. 1. The crossing of the lines may reflect variations in measurement techniques, or may be the result of energy dependence in the electron contamination of the primary beam. Regardless, overall, there is only a small difference in surface dose with nominal beam energy. In contrast, cobalt beams produce a higher surface dose that also increases with field size, ranging between 20%−85% of the $D_{\max}$ dose (Fig. 2).

#### A.2 Beam‐modifying devices

For megavoltage beams, the surface dose increases with the presence of an acrylic or polycarbonate block tray (but not a metal tray^(^ ^22^ ^)^), particularly for larger fields. The relative increase is negligible for a $5\;\;\times \;\;5\;{\text{cm}}^{2}$ field, 10%−20% for a $25\;\;\times \;\;25\;{\text{cm}}^{2}$ field, and ~50% for a $40\;\;\times \;\;40\;{\text{cm}}^{2}$ field.^(^ ^11^ ^,^ ^13^ ^,^ ^22^ ^,^ ^31^ ^,^ ^35^ ^,^ ^38^ ^,^ ^39^ ^)^

Collimating a treatment field with blocks has only a small impact on surface dose (in addition to the impact from the block tray), increasing the relative dose by ~10% at 8 MV and ~25% at 18 MV versus a treatment field defined by jaws alone.^(^ ^31^ ^)^ However, the skin dose underneath a block is substantially reduced.^(^ ^29^ ^)^ Almost no change in skin dose is observed if the treatment field is defined by a multileaf collimator.^(^ ^31^ ^)^

Historically, block trays were not necessarily fixed to the accelerator head; the skin dose increases substantially as the distance between the block tray and the skin surface is reduced. For a cobalt beam, the skin dose (as a fraction of the $D_{\max}$ dose) (D) has been described by:^(^ ^40^ ^)^

(1) $$D=0.25\cdot h^{-0.44}\cdot r^{0.72}$$

with *h* being the separation between the skin and the tray, and *r* being the radius of the equivalent circular field. Treatments with modern linear accelerators predominantly use a fixed block tray position. Although the distance from the block tray would certainly impact the skin dose, no similar equation has been derived, and such a nonstandard treatment would likely be not well described by a general skin dose model such as that proposed here.

Use of a physical wedge tends to decrease surface dose relative to an open field, although this effect is small (generally less than a 20% relative difference, even for 60° wedges); however, this relationship is complicated and not always consistent between studies.^(^ ^14^ ^,^ ^31^ ^)^ In contrast to physical wedges, dynamic wedges have a negligible impact on the surface dose relative to open fields.^(^ ^14^ ^,^ ^31^ ^)^

Field modulation also has little impact on surface dose; studies have found the surface dose to have increased or decreased, but only by an ~10% relative change.^(^ ^15^ ^,^ ^26^ ^,^ ^41^ ^)^

#### A.3 SSD and setup

Surface dose increases slightly as SSD decreases, although this effect is relatively small (~10% relative change with SSD change from 100 cm to either 85 or 120 cm). However, the impact of SSD can exceed 20% for large fields $\left(\geq \;20\;\;\times \;20\;{\text{cm}}^{2}\right)$ when other modifying devices, such as blocks, are involved.^(^ ^31^ ^)^

The presence of a carbon fiber treatment couch increases the skin dose from posterior fields (Table 1).^(^ ^34^ ^,^ ^42^ ^–^ ^44^ ^)^ Even tennis‐string ribbing can slightly increase the skin dose directly under the ribbing.^(^ ^44^ ^)^ In general, the increase in skin dose is more pronounced for cobalt than for megavoltage beams,^(^ ^34^ ^)^ and for small fields as compared to large fields.^(^ ^42^ ^)^

**Table 1:** Surface dose from open fields as compared to fields treated through a carbon fiber couch.

		*Surface Dose* (% of $D_{\max}$ *dose*)	
*Energy*	*Field Size* ($cm^{2}$)	*Open Field*	*Through Couch*
Co‐60	10×10	18	75
6 MV	10×10	21	51
	10×10	20	68
8 MV	20×20	32	80
	40×40	42	84
20 MV	10×10	20	32

Note: 8 MV data from Yu et al.^(^ ^33^ ^)^; other energies from Thomas and Palmer.^(^ ^24^ ^)^

Similarly, immobilization devices such as thermoplastic masks increase the skin dose (e.g., from 16% to 40% of the $D_{\max}$ dose with a lightly stretched mask).^(^ ^14^ ^,^ ^27^ ^,^ ^45^ ^)^ Even patient covering material, such as hospital gowns or blankets, can increase skin dose when they cover the primary field, by up to a 10% absolute difference in clinical situations.^(^ ^46^ ^)^

#### A.4 Obliquity

Oblique beam incidence increases the surface dose. While there is little dosimetric impact from small angles (<40°), the surface dose increases sharply at larger angles, the relative dose being 50% larger at an obliquity of ~55°.^(^ ^23^ ^,^ ^25^ ^)^ There is only small dependence on beam energy,^(^ ^26^ ^,^ ^36^ ^)^ and no impact from field modulation on this increase.^(^ ^15^ ^)^ For a surface at 90° relative to the incident beam, the dose is 58%−65% of the $D_{\max}$ dose for a $10\;\times \;10\;{\text{cm}}^{2}$ field at both 6 MV and cobalt energies.^(^ ^20^ ^,^ ^36^ ^)^

#### A.5 Off‐axis position

Within the treatment field, surface dose is relatively uniform across the field. However, it decreases slightly at the edge of the field (e.g., the outer 2 cm of a $10\;\times \;10\;{\text{cm}}^{2}$ field).^(^ ^29^ ^–^ ^32^ ^)^ This decrease is typically less than a 10% relative decrease, but can approach a 30% relative decrease at the edge of the treatment field in the presence of beam modifiers.^(^ ^31^ ^)^

#### A.6 Exit dose

With megavoltage treatments, substantial skin dose comes from beams exiting the patient. The dose to the exit surface is less than predicted by the percent depth dose (PDD) because of a lack of backscatter material beyond the patient's exit surface. The dose is lower than the PDD by ~15% (relative difference) for 6 MV or cobalt beams, and is not largely different for other megavoltage energies.^(^ ^19^ ^,^ ^26^ ^,^ ^35^ ^,^ ^36^ ^)^ However, approximately full scatter conditions are achieved (i.e., the exit dose is that predicted by the PDD) with minimal material, including virtually any immobilization device, behind the patient.^(^ ^19^ ^,^ ^35^ ^,^ ^36^ ^)^

#### A.7 Outside the treatment field

**Figure 3:**
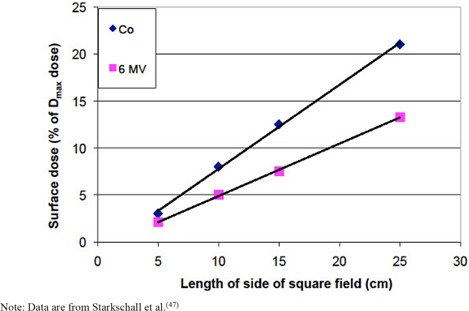
Surface dose (as a percentage of the $D_{\max}$ dose) 2 cm from the field edge for a Co‐60 beam and a 6 MV beam. Dose is shown as a function of the size of the edge of the equivalent square field. Note: Data are from Starkschall et al.^(^ ^47^ ^)^

The out‐of‐field surface doses from cobalt and 6 MV beams are between ~2% and ~20% of the $D_{\max}$ dose at 2 cm from the field edge,^(^ ^29^ ^,^ ^47^ ^)^ being larger for larger treatment fields and for cobalt beams (Fig. 3).

**Figure 4:**
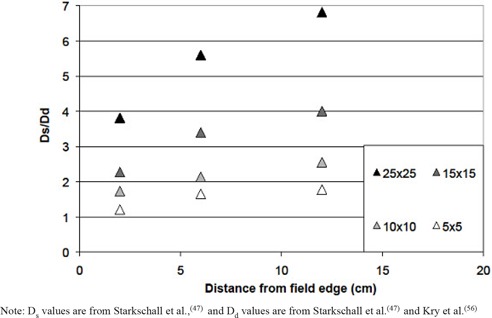
Ratio of the surface dose ($D_{s}$) to the dose at depth ($D_{d}$; taken at 0.5 cm depth) as a function of the distance from the edge of the treatment field for Co‐60 beams with various field sizes. Note: $D_{s}$ values are from Starkschall et al.,^(^ ^47^ ^)^ and $D_{d}$ values are from Starkschall et al.^(^ ^47^ ^)^ and Kry et al.^(^ ^56^ ^)^

**Figure 5:**
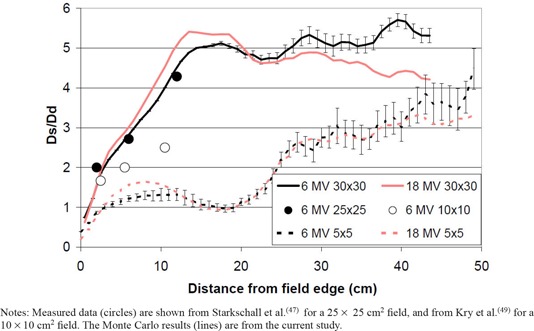
Ratio of the surface dose ($D_{s}$) to the dose at depth ($D_{d}$; taken at 5 cm depth) as a function of the distance from the edge of the treatment field for 6 MV and 18 MV beams for various field sizes. Error bars are included on the 6 MV data to illustrate the statistical uncertainties in the calculated values; similar uncertainties exist in the 18 MV data, but are excluded for visual clarity. Notes: Measured data (circles) are shown from Starkschall et al.^(^ ^47^ ^)^ for a $25\;\times \;25\;{\text{cm}}^{2}$ field, and from Kry et al.^(^ ^49^ ^)^ for a $10\;\times \;10\;{\text{cm}}^{2}$ field. The Monte Carlo results (lines) are from the current study.

At greater distances, less data are available regarding surface dose. It has been observed that out‐of‐field dose varies minimally with depth^(^ ^48^ ^,^ ^49^ ^)^ except near the surface where it is highly elevated;^(^ ^32^ ^,^ ^48^ ^–^ ^50^ ^)^ surface doses are a factor of 2–7 greater than the dose a few centimeters below the surface. The dose at depth (dose a few cm below the surface) decreases approximately exponentially as the distance from the field edge increases, and is approximately 1% of the $D_{\max}$ dose at 10 cm from the field edge.^(^ ^48^ ^)^ The ratio of the surface dose to this dose at depth (taken as the dose at 0.5 cm depth for cobalt and 5 cm depth for megavoltage beams) is shown in Fig. 4 for cobalt beams. The ratio of surface dose to dose at depth is larger for greater distances from the field edge and larger field sizes. A similar trend can be seen for megavoltage beams in Fig. 5.

### B. Additional studies

This section outlines our additional studies to assess surface doses.

#### B.1 Cobalt beam with block tray

##### B.1.1 Methods

We investigated the effect of a modern block tray attached to the gantry head on surface dose for a cobalt radiotherapy unit. Dose was measured with 5 mm×5 mm×0.4 mm thick flat packs of LiF thermoluminescence dosimeter powder (TLD‐100; Quantaflux, Dayton, OH), which have an effective depth of measurement of 0.44 mm (based on their relative electron density of 2.2).^(^ ^51^ ^)^ This thickness of TLD would slightly overestimate the true surface dose by ~15% of the local dose,^(^ ^14^ ^,^ ^28^ ^,^ ^41^ ^)^ which was considered acceptable error for this study because of the large interstudy variability of surface dose measurements (e.g., Fig. 2).

The batch of TLD powder used in this study was previously calibrated with a cobalt‐60 source. The experimental TLD were corrected according to the batch characterization for both fading and linearity. These TLDs were evaluated by Radiation Dosimetry Services (RDS) according to a previously detailed procedure, and with an uncertainty (at a 90% confidence level) of ± 3.9%.^(^ ^52^ ^)^

Three TLD flat packs were simultaneously irradiated on the surface of a rectangular solid water phantom, as well as at $d_{\max}$, within 5 × 5 and $30\;\times \;30\;{\text{cm}}^{2}$ fields, with and without an acrylic block tray.

##### B.1.2 Results

The impact on the skin dose of a block tray in a cobalt beam is shown in Table 2. For a small field, there was no increase in the skin dose, consistent with megavoltage beams.^(^ ^31^ ^)^ However, for the large field, the skin dose decreased slightly (a 10% relative reduction) when the block tray was added. This is in contrast to megavoltage beams, for which the surface dose increased substantially, and is likely the result of the softer cobalt spectrum. In general, cobalt beams have a higher surface dose than megavoltage beams because of the lower energy of scattered photons and abundant secondary electrons. The lower energy spectrum of the scattered photons appears to result in a net attenuation of electrons and low‐energy photons in the block tray (and, hence, a decrease in the surface dose), in contrast to megavoltage beams for which the block tray results in a net production of electrons.^(^ ^31^ ^)^

**Table 2:** Skin dose from a cobalt beam for two field sizes as influenced by the presence of a block‐tray.

	*Skin Dose (% of* $D_{\max}$ *dose*)	
*Field Size* $\left(cm^{2}\right)$	*No Tray*	*Tray*
5×5	31	32
30×30	75	68

#### B.2 Out‐of‐field surface dose

##### B.2.1 Methods

As distance from the field edge increases, the out‐of‐field dose at depth decreases to ~1% of the $D_{\max}$ dose at 10 cm from the field edge, and to ~0.05% of the $D_{\max}$ dose at 50 cm from the field edge. The surface dose outside the treatment field at each of these distances is a factor of 2–7 greater than the dose a few centimeters below the surface (where the dose becomes relatively constant with depth). This increase is dependent on field size and distance from the field edge, but these dependencies have not been well studied. Therefore, we conducted Monte Carlo simulations using a model of a Varian 2100 linac (Palo Alto, CA) developed in MCNPX,^(^ ^53^ ^)^ and previously benchmarked (including doses near the surface).^(^ ^49^ ^,^ ^50^ ^)^

For simulated 6 MV and 18 MV beams, the dose to the surface of a water tank (at 0.5 mm depth) was calculated based on the electron energy deposition over the most superficial 1 mm of water. Although not strictly at the surface, this depth offered a reasonable compromise between computational accuracy and dosimetric accuracy, particularly as the dose gradient is not as steep near the surface for out‐of‐field locations.^(^ ^49^ ^)^ To achieve sufficient precision, the 1 mm thick tally was 4 cm wide by 1 cm long. This surface dose was calculated up to 60 cm from the central axis for 5 × 5 and $30\;\times \;30\;{\text{cm}}^{2}$ fields. The dose was also calculated throughout the phantom so that the surface dose relative to the dose at depth could be readily determined. Two billion histories were run for each simulation, ensuring a reasonable degree of statistical certainty (5% on average at 1 sigma) in each tally.

##### B.2.2 Results

The ratio of the surface dose to the dose at depth at a given distance from the field edge varied little between the 6 MV and 18 MV beams (Fig. 5). Moving away from the edge of the treatment field, the surface dose ratio increased and remained approximately constant at large distances from the field edge. For the $5\;\times \;5\;{\text{cm}}^{2}$ field the surface dose ratio increased to approximately 3, while for the $30\;\times \;30\;{\text{cm}}^{2}$ field the surface dose ratio increased to approximately 5. The Monte Carlo simulated surface dose ratios were consistent with previously measured values for a 6 MV beam (Fig. 5).

## III. GENERAL DOSIMETRY SYSTEM

### A.1 Formalism

In this section, guidelines are provided for general dosimetry estimates of skin dose (i.e., surface dose). In creating a general system, high patient‐specific accuracy is unachievable, and is often unnecessary. Previously developed general dosimetry systems (such as the AAPM TG‐36 data,^(^ ^48^ ^)^ or Peridose software^(^ ^54^ ^,^ ^55^ ^)^) typically offer a local accuracy of approximately ±30%.^(^ ^55^ ^,^ ^56^ ^)^ Therefore, we considered local dosimetric accuracy of ±30% optimal.

### A.2 Energy, field size, and beam‐modifying devices

Skin dose on the entrance surface within a treatment field was primarily determined by field size and the presence or absence of a block tray. For megavoltage beams, skin dose from any energy was calculated as the average of the best‐fit lines in Fig. 1 modified by a tray factor (TF) term. This term increased with field size, from 0% for a 0 × 0 field to 50% for a $40\;\times \;40\;{\text{cm}}^{2}$ field. Mathematically, the skin dose ($D_{s}$) versus the $D_{\max}$ dose ($D_{m}$) was calculated within a megavoltage field as:

(2) $$D_{s}=\left(\frac{D_{m}}{100}\right)[6.63+0.926\cdot FS+0.5\cdot FS\cdot TF]$$

where *FS* is the size of the equivalent square field (in cm, at the isocenter plane), and *TF* is 1 if a tray is present and 0 if there is no tray. The skin dose within a cobalt field was similarly described by the best‐fit line in Fig. 2, but was not impacted by a block tray.

(3) $$D_{s}=\left(\frac{D_{m}}{100}\right)[12.9+1.85\cdot FS]$$

Variations in block tray position relative to the skin does impact the skin dose and could be accounted for in Eq. (1); however, this separation is not typically reported in historical patient records, and was therefore neglected here. For dosimetric evaluation within the collimator opening but under a block, it was assumed that the point of interest would receive a dose similar to that received 2 cm outside the treatment field defined by the jaws. Dosimetry for such a point is described in Section A.6 below. This assumption is physically reasonable and is supported by the literature; Butson et al.^(^ ^29^ ^)^ measured surface doses of 20%−23% of the $D_{\max}$ dose under a block, while the dosimetry in III.A.6 predicted a dose of 20% for the same condition.

If the beam intercepts the treatment couch, the skin dose could be substantially increased. Historically, patients were not treated through the couch, unless a tennis racket style insert was used. For the purpose of an epidemiologic study therefore, we ignored the couch's impact in this dosimetry system. However, as illustrated in Table 1, if the treatment fields are directed through the treatment couch, it would not be appropriate to ignore this effect. Similarly, immobilization devices and patient coverings may substantially increase the skin dose and may need to be accounted for. Due to the lack of documentation on such devices in historical patient records, this becomes an area of increased uncertainty in surface dose estimates for retrospective evaluation.

### A.3 Obliquity

Skin dose increases substantially for large oblique angles of incidence. Although this increase is monotonic (but nonlinear) with increasing angle, it is difficult from patient records to determine the angle between the beam and the skin at a particular point. Therefore, our model simply assumed either a normal surface or a surface lateral to the beam incidence (i.e., 900 obliquity/tangential orientation). In the case of a lateral surface, the dose from either a megavoltage or cobalt beam was taken as 65% of the $D_{\max}$ dose. This approach will overestimate the dose to surfaces at large but nonorthogonal angles to the incident beam, and is in general associated with greater uncertainty in the dose estimate.

### A.4 Other treatment parameters

Off‐axis position and SSD do have some effect on the skin dose, but these effects were ignored in our model because of their complex but minimal impact on the skin dose. For most scenarios, these treatment parameters made less than a 10% local change. However, this was not always the case. The SSD, in particular, when combined with other beam modifiers, could induce changes in the surface dose of > 20%. In general, the more complex a given scenario, the more uncertainty will exist with dose estimates based on a general model such as that proposed here.

### A.5 Exit dose

The lack of backscatter material results in an ~15% relative reduction in dose at an exit surface. Although this dose perturbation is relatively small, it is easily accounted for. The skin dose at any exit surface from cobalt or megavoltage beams was taken as the expected dose from the PDD multiplied by 0.85. This applied to the exit dose within the treatment field $\left(D_{s}=D_{m}\times \text{PDD}\times 0.85\right)$. It also applied to the exit dose under a block $\left(D_{s}=\text{block transmission}\times D_{m}\times 0.85\right)$, where the block transmission (as dose in full phantom) was 8%.^(^ ^57^ ^)^ It also applied to the dose at any exit surface outside the treatment field ($D_{s}=\text{dose}$ predicted at depth outside the treatment field × 0.85). The dose predicted at depth outside the treatment field was calculated with generic out‐of‐field data.^(^ ^57^ ^)^

### A.6 Out‐of‐field dose

Near the treatment field (~2 cm from the field edge), the entrance surface dose can be described in terms of the $D_{\max}$ dose by the best‐fit lines shown in Fig. 3 for megavoltage or cobalt beams. For megavoltage beams, it was assumed that the block tray would affect the out‐of‐field skin dose in the same manner as it did within the treatment field (Eq. (2)), resulting in the following equation:

(4) $$D_{s}=\left(\frac{D_{m}}{100}\right)[-0.686+0.557\cdot FS+0.27\cdot FS\cdot TF]$$

while for cobalt beams:

(5) $$D_{s}=\left(\frac{D_{m}}{100}\right)[-1.17+0.894\cdot FS]$$

For distances beyond ~2 cm from the field edge, the skin dose was first described relative to the dose at depth (i.e., any depth deeper than a few cm). The dose at depth was calculated for a given distance from the field edge with previous general out‐of‐field dose data,^(^ ^57^ ^)^ although several such collections of out‐of‐field dose data are available and could be used, such as the AAPM TG‐36 data.^(^ ^48^ ^,^ ^55^ ^)^

The ratio of the surface dose to the dose at depth $\left(D_{s}{/D}_{d}\right)$ is presented in Figs. 4 and 5. A general form for this ratio as a function of distance from the field edge (χ, in cm) and field size (*FS*, in cm) was estimated based on Figs. 4 and 5 by assuming that the dose ratio increased linearly as a function of field size until 12 cm from the field edge, beyond which it was assumed to be constant. For small fields ($5\;\times \;5\;{\text{cm}}^{2}$), the surface dose ratio $\left(D_{s}{/D}_{d}\right)$ increased to a maximum of approximately 2 for both megavoltage and cobalt fields. From Fig. 4, this will overestimate the dose between 10–25 cm from the field edge (where $\left(D_{s}{/D}_{d}\right)$ is actually < 2), but will underestimate the dose at distances > 25 cm (where $\left(D_{s}{/D}_{d}\right)$ is < 2). For large fields ($40\;\times \;40\;{\text{cm}}^{2}$), the surface dose ratio increased to a maximum of 5 for megavoltage fields and 7 for cobalt fields, and is well described over all distances from the field edge. Mathematically, the surface dose ratio was described as:

(6) $$\frac{D_{s}}{D_{d}}=(FS+10)\left(\frac{x}{150}\right)+\left(\frac{FS}{100}\right)+1$$

for megavoltage beams, and

(7) $$\frac{D_{s}}{D_{d}}=\left(\frac{FS\cdot x}{85}\right)+\left(\frac{FS-3}{10}\right)+1$$

for cobalt beams. As the surface dose ratio is approximately constant at distances greater than 12 cm, χ takes a value of 12 for any greater distances.

**Figure 6:**
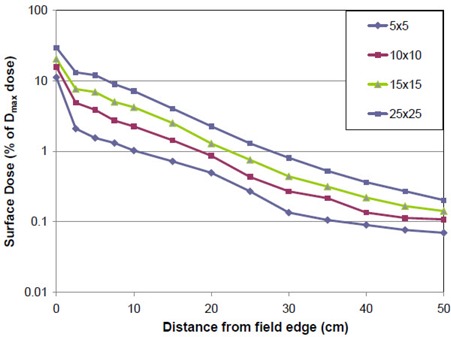
Surface doses outside of the treatment field for MV beams. Plots are the AAPM TG‐36 data^(^ ^48^ ^)^ scaled by the surface dose ratios of Eq. (6). Data is plotted for four different field sizes.

Out‐of‐field surface doses as a percent of the $D_{\max}$ dose are presented in Fig. 6 for megavoltage beams. This figure shows the AAPM TG‐36 data^(^ ^48^ ^)^ scaled by the surface dose ratio (Eq. (6)) versus field size and distance from the field edge. At the field edge the surface dose is the in‐field value (Eq. (2)), while at 2 cm from the field edge the surface dose is based on Eq. (4). Of note, the surface dose could exceed 10% of the $D_{\max}$ dose for large fields, even relatively far from the field edge.

It was assumed that the block tray and other treatment parameters (such as SSD) did not have any impact on the out‐of‐field skin dose at distances beyond 2 cm from the field edge. While it is likely that these assumptions are not completely valid, it is also likely that any errors introduced by them are relatively small compared to the uncertainty inherent in Eqs. (6) and (7), and generally less relevant given the smaller magnitude of the doses outside the beam.

### B. Validation of general surface dosimetry system

#### B.1 Methods

A collection of surface doses were measured and compared to calculations with the dosimetry system developed in Section A. General dosimetry system above. Measurements were made with flat‐pack TLD (as in Section B.1.1 Methods above) at several locations on anthropomorphic phantoms, with 2 TLD irradiated and averaged at each location for each of an AP and PA field. The first irradiation used a pediatric six‐year‐old Alderson phantom irradiated with a $15\;\times \;15\;{\text{cm}}^{2}$ Co‐60 field, using a block tray in the head of the machine (AECL Therac‐80). The phantom was positioned at 80 cm SSD, and was irradiated with identical anterior and posterior beams. The dose was measured at 6 locations: near the center of the field (on the anterior and posterior surfaces), under the block, inside the treatment field but on a lateral phantom surface (tangential exposure), and at 2 and 10 cm outside of the treatment field (relative to the anterior field). For the out‐of‐field locations, the spectrum is softer, requiring an additional energy correction for TLD measurements.^(^ ^58^ ^)^ Energy correction factors were taken from Scarboro et al.,^(^ ^58^ ^)^ with the same relative correction factor used for both cobalt and 6 MV irradiations (this is an approximation for cobalt for which no specific data exists, and the uncertainty in the cobalt dose measurements is correspondingly increased). The dose was also calculated at each location for each of the anterior and posterior fields. In particular, at the center of the field, the dose (% of $D_{\max}$) from the entrance field was calculated from Eq. (3), and the dose from the exit field was calculated as $D_{m}\times \text{PDD}\times 0.85$ from Section III.A.5. For the measurement location under the block, the dose from the entrance (anterior) field was calculated from Equation 5 and the dose from the exit (posterior) field was calculated from Section III.A.5 $\left(D_{s}=\text{block transmission}\times D_{m}\times 0.85\right)$. For the lateral surfaces, the dose from each of the anterior and posterior fields was taken as 65% of the given dose. For the location 2 cm from the field edge, the entrance dose was calculated by Eq. (5). Because of beam divergence, this location was actually on the edge of the posterior treatment field (rather than 2 cm outside it), and the dose from this field was calculated as half of the in‐field exit dose: $0.5\times D_{m}\times \text{PDD}\times 0.85$. For the location 10 cm from the field edge, the entrance dose (from the anterior field) was calculated as the scaling factor (Eq. (7)) multiplied by the dose estimated at depth.^(^ ^48^ ^)^ The exit dose (posterior field) was the dose estimated at depth^(^ ^48^ ^)^ multiplied by 0.85 (accounting for the lack of backscatter).

A second irradiation used an adult anthropomorphic phantom irradiated with 6 MV (Varian Medical Systems, Palo Alto, CA) anterior and posterior $30\;\times \;30\;{\text{cm}}^{2}$ mantle fields with lung blocks. The dose was measured and calculated within the unblocked treatment field (anterior and posterior surfaces), at 2 locations under the blocks, at 2 locations on a lateral surface, 2 cm from the field edge, 5 cm from the field edge, and 15 cm from the field edge.

#### B.2 Results

For the cobalt irradiation of the pediatric phantom, the calculated dose from each of the anterior and posterior fields to each of the measurement locations is shown in Table 3, along with the measured value.

**Table 3:** Skin dose to measurement locations from cobalt irradiation of a pediatric phantom. Doses (from the anterior and posterior fields) are shown as compared to the measured doses. The local percent difference between each measured and calculated dose is also shown.

		*Skin Dose (% of* $D_{\max}$ *dose*)					
			*Anterior Field*			*Posterior Field*	
*Phantom Surface*	*Measurement Location*	*Calculated*	*Measured*	*Difference (%)*	*Calculated*	*Measured*	*Difference (%)*
Anterior	In‐field	41	40	0.5	39	37	5.1
	Under block	12	14	−10	6.8	9.7	−30
	2 cm outside field	12	10	18	19	16	24
	10 cm outside field	7.9	4.2	88	1.7	1.1	58
Lateral	In‐field	65	59	11	65	66	1.3
Posterior	In‐field	39	35	11	41	45	−10

Overall, the average magnitude of local difference between the calculated and measured doses was 22%. In general, the agreement was very good, except at very small doses where the differences could be more substantial. The average difference in terms of percent of the $D_{\max}$ dose, which downplays the importance of large differences in small doses, was less than 3%.

Similarly, the calculated and measured doses are shown in Table 4 for the 6 MV adult irradiation. The average magnitude of local difference between the calculated and measured doses was 21%. The largest differences were seen in high‐dose gradient locations (on a lateral surface) and at low doses. The average difference as a percent of the $D_{\max}$ dose was less than 5%.

**Table 4:** Skin dose to measurement locations from 6 MV mantle irradiation of an anthropomorphic phantom. Doses (from the anterior and posterior fields) are shown as compared to the measured doses. The local percent difference between each measured and calculated dose is also shown.

		*Skin Dose (% of* $D_{\max}$ *dose*)					
			*Anterior Field*			*Posterior Field*	
*Phantom Surface*	*Measurement Location*	*Calculated*	*Measured*	*Difference (%)*	*Calculated*	*Measured*	*Difference (%)*
Anterior	In‐field	49	48	*2.4*	35	28	*22*
	Under block (1)	24	26	−6.6	6.8	8.5	−20
	Under block (2)	24	20	*19*	6.8	10.2	−33
	2 cm outside field	24	20	*20*	18	24	−25
	5 cm outside field	16	10	*60*	5.5	4.0	*38*
	15 cm outside field	5.0	4.4	*13*	0.9	0.8	*19*
Lateral	In‐field (1)	65	56	*16*	65	66	−2.1
	In‐field (2)	65	46	*40*	65	59	*11*
Posterior	In‐field	44	42	*3.2*	49	61	−19

The uncertainty in the TLD measurements varied between measurement locations. The underlying uncertainty in the TLD calibration protocol was less than 4% (at 1 sigma).^(^ ^52^ ^)^ However, for out‐of‐field locations, the energy correction factor introduced additional uncertainty, on the order of 2%–3% for the 6 MV beam (for which approximate energy correction factors were available), and potentially up to 5% or more for the cobalt beam.^(^ ^57^ ^)^ In‐field dose measurements would tend to be overestimated because of the buildup of the flat‐packs (as compared to the surface dose), by up to 15% relative dose for small MV photon fields, but by less for large or cobalt fields. Similarly, exit dose measurements would also tend to be overestimated because the thickness of the TLD would partially recover the 15% lost exit dose. Usually, the uncertainty in the TLD was less than the differences between the measurement and the simple skin dose model. That is, the percent differences in Tables 3 and 4 were generally dominated by the simplification of treatment parameters that went into the model, rather than uncertainty in the measurements.

Although there could be sizable error in individual dose calculation points, overall the general dosimetry system developed in Section A above was reasonably accurate and broadly applicable to the calculation of skin dose. The agreement with measurement of this skin model was comparable to the model proposed by Lamb and Blake^(^ ^18^ ^)^ who found agreement within 5% of the $D_{\max}$ dose. However, the current model also offers skin dose estimates outside of the treatment field and for more treatment energies.

## IV. CONCLUSIONS

In this study, we reviewed the literature and conducted our own measurements to describe the relationship between treatment parameters and the radiotherapy dose to the patient's skin. There was little difference in skin dose between MV beam energies, but substantially different doses resulted from cobalt beams. The skin dose within the treatment field increased substantially with increasing field size, and, for MV beams, increased further in the presence of a block tray. The skin dose also increased as obliquity between the incident photon beam and the patient surface increased. Outside of the treatment field, the skin dose was highly elevated relative to the dose at depth in the patient, by as much as a factor of 7. This increase in the out‐of‐field skin dose was maximized for large fields and large distances from the field edge, and was slightly greater for cobalt beams than for MV beams.

In this work, we also developed a broadly applicable dosimetry system that provides an estimate of the skin dose from radiotherapy treatments. Compared to measurements, this dosimetry system was generally accurate within 21% (local difference), or 4% of the $D_{\max}$ dose, although individual skin locations or treatments could have much larger local differences. While this dosimetry system was designed for calculating skin dose for epidemiology studies, it could be generally applied to a wide range of clinical situations.

## ACKNOWLEDGMENTS

Supported by Grants No. 6‐39403‐GI from the Lance Armstrong Foundation, Austin, TX, and U24CA057227 from the National Cancer Institute, Bethesda, MD.
